# Influence of Social Support, Financial Status, and Lifestyle on the Disparity Between Inflammation and Disability in Rheumatoid Arthritis

**DOI:** 10.1002/acr.24996

**Published:** 2022-12-28

**Authors:** James M. Gwinnutt, Sam Norton, Kimme L. Hyrich, Mark Lunt, Bernard Combe, Nathalie Rincheval, Adeline Ruyssen‐Witrand, Bruno Fautrel, Daniel F. McWilliams, David A. Walsh, Elena Nikiphorou, Patrick D. W. Kiely, Adam Young, Jacqueline R. Chipping, Alex MacGregor, Suzanne M. M. Verstappen

**Affiliations:** ^1^ University of Manchester Manchester UK; ^2^ King's College London London UK; ^3^ University of Manchester and Manchester University NHS Foundation Trust Manchester UK; ^4^ University of Montpellier Montpellier France; ^5^ Hôpital Purpan and Université Toulouse III‐Paul Sabatier University Toulouse France; ^6^ Sorbonne University, Assistance Publique Hôpitaux de Paris, Pitie Salpetriere Hospital, and Pierre Louis Institute of Epidemiology and Public Health Paris France; ^7^ University of Nottingham and NIHR Nottingham Biomedical Research Centre Nottingham UK; ^8^ University of Nottingham and NIHR Nottingham Biomedical Research Centre, Nottingham, UK, Sherwood Forest Hospitals NHS Foundation Trust Sutton‐in‐Ashfield UK; ^9^ King's College and King's College Hospital London UK; ^10^ St. George's University Hospitals NHS Foundation Trust and St. George's University of London London UK; ^11^ University of Hertfordshire Hatfield UK; ^12^ University of East Anglia and Norfolk and Norwich University Hospitals NHS Trust Norwich UK

## Abstract

**Objective:**

To investigate how social support, financial status, and lifestyle influence the development of excess disability in rheumatoid arthritis (RA).

**Methods:**

Data were obtained from the Étude et Suivi des Polyarthrites Indifférenciées Récentes (ESPOIR) cohort study of people with RA. A previous analysis identified groups with similar inflammation trajectories but markedly different disability over 10 years; those in the higher disability trajectory groups were defined as having “excess disability.” Self‐reported data regarding contextual factors (social support, financial situation, lifestyle) were obtained from participants, and they completed patient‐reported outcome measures (pain, fatigue, anxiety, depression) at baseline. The direct effect of the contextual factors on excess disability and the effect mediated by patient‐reported outcome measures were assessed using structural equation models. Findings were validated in 2 independent data sets (Norfolk Arthritis Register [NOAR], Early Rheumatoid Arthritis Network [ERAN]).

**Results:**

Of 538 included ESPOIR participants (mean age ± SD 48.3 ± 12.2 years; 79.2% women), 200 participants (37.2%) were in the excess disability group. Less social support (β = 0.17 [95% confidence interval (95% CI) 0.08, 0.26]), worse financial situation (β = 0.24 [95% CI 0.14, 0.34]), less exercise (β = 0.17 [95% CI 0.09–0.25]), and less education (β = 0.15 [95% CI 0.06, 0.23]) were associated with excess disability group membership; smoking, alcohol consumption, and body mass index were not. Fatigue and depression mediated a small proportion of these effects. Similar results were seen in NOAR and ERAN.

**Conclusion:**

Greater emphasis is needed on the economic and social contexts of individuals with RA at presentation; these factors might influence disability over the following decade.

**SIGNIFICANCE & INNOVATIONS**

- Financial instability and less social support, education, and exercise were associated with disability in rheumatoid arthritis (RA), independent of inflammation level.
- Patient‐reported outcomes (pain, fatigue, anxiety, depression) only mediated a small proportion of this effect.
- Social and economic factors play a key role in explaining the inflammation–disability gap evident in long‐term outcomes in RA; potentially people at risk of excess disability would benefit from greater assessment (e.g., via remote technologies), signposting to community groups, and targeted nonpharmacologic interventions (e.g., exercise).

## INTRODUCTION

Antecedent factors contribute to the progression of chronic illness. The Dahlgren‐Whitehead model of health determinants highlights the multi‐layered nature of these social determinants of health whereby living and working conditions, including education, employment, and housing (1) influence social and community networks (2), which in turn can influence individual lifestyle factors (e.g., smoking, exercise, weight, and alcohol use). These individual, contextual, and societal factors are important components determining the onset and progression of chronic illness (e.g., diabetes, cardiovascular disease) alongside biologic determinants such as genetic factors, potentially by moderating long‐term stress levels (3).

Rheumatoid arthritis (RA) is a chronic condition involving the inflammation of synovial joints, potentially leading to long‐term pain and functional disability (4, 5). Improvements in available treatments for RA mean that inflammation can be controlled at low levels into the long‐term for the majority of people, but for many individuals this does not correlate with improvements in disability (the so‐called “inflammation–disability gap” in RA) (6, 7). Our previous analysis of 3 large‐scale European cohorts of individuals with early RA demonstrated that there are trajectory groups that share similar inflammation trajectories over 10 years of follow‐up but have markedly different disability trajectories (8). The level of disability in these trajectory groups was relatively fixed from baseline, indicating that factors prior to the onset of RA may influence baseline disability and thus subsequent disability.

In a previous analysis of 2 RA cohorts exploring longitudinal trajectories of functional impairment, lower socioeconomic status (defined using the UK's Index of Multiple Deprivation) predicted increased disability over time (9). A study from Texas reported that lower socioeconomic status (composite measure comprised of education, income, and occupation) was associated with increased disease activity, erosions, and functional disability (10). A study of Swedish mortality records reported lower education was associated with 2‐fold increased risk of death in individuals with RA (11). Lifestyle factors, such as smoking, exercise, body weight, and alcohol consumption have all been reported to influence outcomes in RA (12, 13, 14). Furthermore, lower social support is correlated with more depression, distress, and disability in RA (15, 16, 17). Traumatic life events, such as the death of a spouse, may also influence outcomes (18). In summary, disparities in socioeconomic status, lifestyle, and social support may explain part of the aforementioned inflammation–disability gap in RA. However, these factors are typically studied in isolation, meaning the relative contributions of economic, social, and lifestyle factors on RA outcomes are unclear.

Therefore, the aim of this study was to determine the association between specific social determinants of health (rather than deprivation indices) and antecedent events prior to the onset of RA and inflammation–disability trajectory group membership (8). Furthermore, previous research has shown that pain, fatigue, and depression are strongly associated with excess disability group membership (8). Therefore, a second objective was to explore the mediating effect of patient‐reported outcome measures on the relationship between these antecedent factors and excess disability in RA.

## PATIENTS AND METHODS

The data for this analysis were obtained from the Étude et Suivi des Polyarthrites Indifférenciées Récentes (ESPOIR) study, a cohort of individuals with inflammatory arthritis recruited from 14 centers across France from 2002 to 2005 (ClinicalTrials.gov identifier: NCT03666091). The ESPOIR inclusion criteria were >2 swollen joints for >6 weeks and <6 months, clinical diagnosis of RA as certain or possible, ages 18–70 years, and no disease‐modifying antirheumatic drugs or glucocorticoids for >2 weeks (19). The ESPOIR cohort study was approved by the Ethics Committee of Montpellier (reference no. 020307).

### Social context, financial situation, and lifestyle variables

Participants of ESPOIR answered several questions at baseline from the Evaluation de la Précarité et des Inégalités de santé dans les Centres d'Examens de Santé (20, 21) questionnaire related to availability of social support (yes/no), including whether participants felt they had someone to rely on for accommodation (accommodation help) or financial assistance (financial help), whether they were married or cohabiting compared with being single, divorced, or widowed (married/cohabiting), and whether they had seen their family in the previous 6 months (family contact). Participants also reported the number of inhabitants of the town or city where they lived (<5,000 inhabitants, 5,000–20,000 inhabitants, 20,000–50,000 inhabitants, >50,000 inhabitants).

The financial situation of participants at baseline was assessed using questions asking participants to self‐report their monthly family income (<€610, €610–1,220, €1,220–1,830, €1,830–2,440, €2,440–2,745, >€2,745), personal income (using the same categories), and whether participants were homeowners (homeowner), had been to a in the previous 6 months (show/cinema), and had been on holiday in the previous 6 months (holiday). Participants also reported their working status (full‐time, part‐time, at home, disabled, student, retired, unemployed, long‐term illness, other) and job level (coded into 3 levels: low‐level [farmer, artisan/trader, worker/laborer, without profession], mid‐level [employee, intermediate occupation], high‐level [management, self‐employed]).

Baseline lifestyle data available in ESPOIR included smoking status (current smoker, yes/no), alcohol consumption (yes/no), body mass index (BMI; calculated from height and weight measured at baseline), and whether participants had participated in sport in the previous 6 months (yes/no). Furthermore, participants reported their education level at baseline (primary, qualifications at 16 years, qualifications at 18 years, undergraduate degree, postgraduate degree). Last, participants reported whether they had experienced any traumatic events or the death of someone close in the 6 months prior to RA onset.

### Clinical variables and patient‐reported outcome measures

At baseline, researchers completed 28 swollen and tender joint counts for each participant, and a blood sample was collected from which C‐reactive protein (CRP) level was measured. ESPOIR participants also completed pain, fatigue, and global assessment visual analog scales (VAS, 1–100 mm) and the French version of the Health Assessment Questionnaire (HAQ), a measure of functional disability (22). Anxiety and depression were assessed using 5 variables from the French version of the Arthritis Impact Measurement Scales 2 (23). The Disease Activity Score in 28 joints (DAS28), a composite measure of disease activity, was calculated from swollen and tender joint counts and CRP level (24). The 2‐component DAS28 (DAS28‐2C), a composite measure of inflammation, was calculated from the swollen joint count and CRP level of individuals (25).

### Excess disability group membership

Our previous analysis of 3 European cohort studies (including ESPOIR) analyzing the trajectories of inflammation (measured using the DAS28‐2C [25], chosen to isolate the influence of inflammation specifically [the target of pharmacologic treatment in RA], as opposed to disease activity [which is likely influenced by inflammation level plus multiple other factors]) and disability (measured using the HAQ [22, 26]) over 10 years identified 5 subgroups (i.e., 1 trajectory with very low inflammation and disability, 2 trajectories with similar low levels of inflammation but one with higher disability than the other, and 2 trajectories with similar high levels of inflammation, again with one group having higher disability than the other [Supplementary Figure 1, available on the *Arthritis Care & Research* website at http://onlinelibrary.wiley.com/doi/10.1002/acr.24996]). The people in these high HAQ groups (i.e., the low inflammation–high HAQ group and the high inflammation–high HAQ group) were described as having “excess disability” in relation to their inflammation level (8). For the current analysis, the 2 subgroups characterized by excess disability were combined and compared with the groups of individuals who had similar inflammation over follow‐up, but lower disability (i.e., the outcome for this analysis is excess disability compared with other individuals with RA with similar inflammation levels but lower disability).

### Validation data sets

The Norfolk Arthritis Register (NOAR) (27) and the Early Rheumatoid Arthritis Network (ERAN) (28) data sets acted as validation data sets. In NOAR, data on current employment status were collected (categorized as working [working], not working [unemployed, off sick, house‐person/parent, retired early–health grounds], retired [retired]) and job seniority level (categorized as low [partly skilled, unskilled], medium [non‐manual skilled, manual skilled], high [professional, managerial and technical]). In ERAN, employment status was available (using the same coding as NOAR), as were deciles of the 2007 Index of Multiple Deprivation (IMD), an area‐level index of deprivation combining income deprivation, employment deprivation, health deprivation and disability, education deprivation, crime deprivation, barriers to housing and services deprivation, and living environment deprivation (29). Ethics approval for NOAR and ERAN came from the Cambridgeshire and Hertfordshire Research Ethics Committee (approval no. 15/EE/0076) and the Trent Research Ethics Committee (approval no. 01/4/047), respectively. Written informed consent was obtained from participants in all 3 cohorts. Data are available upon reasonable request from the principal investigators of each of the 3 data sets (AM [NOAR], DAW [ERAN], BC [ESPOIR]).

### Statistical analysis

The baseline demographic and clinical characteristics of the cohorts were described using descriptive statistics. The associations between each antecedent factor (social support, financial situation and lifestyle) with membership in the excess disability subgroup were assessed using logistic regression, controlling for age and sex. However, many of these variables were correlated. Therefore, a structural equation modeling (SEM) approach was used. Using SEM has several advantages: multiple indicators of an underlying, potentially unmeasurable concept (e.g., social support) can be combined to produce latent variables (closer approximations of these underlying constructs). Furthermore, the effect of these latent variables on excess disability can be broken down into direct effects and indirect effects where antecedent factors influence disability via intermediary variables (i.e., mediation analysis), allowing for a greater understanding of the pathways from these antecedent factors to excess disability in RA.

Initially, latent variables were constructed summarizing the social support participants received (measured using accommodation help, financial help, family contact, and married/cohabiting variables) and the financial situation of participants (measured using family income, personal income, homeowner status, show/cinema visits, holiday, working status, and job level variables); these latent variables were assessed using confirmatory factor analysis (maximum likelihood estimator), with model fit assessed using the Tucker‐Lewis Index (TLI) (good fit >0.9) and the root mean square error of approximation (RMSEA) (good fit <0.08). Several of the ordinal variables were dichotomized to aid model convergence: family income (≥€1,830 versus <€1,830; dichotomized at the middle category), personal income (≥€1,830 versus <€1,830), and working status (full‐time and part‐time versus other categories). The total effects each of these latent variables, plus lifestyle variables (smoking, alcohol, BMI, exercise) and education, had on membership in the excess HAQ group were assessed using SEM (4 models in total).

To investigate the mediating effect of pain, fatigue, anxiety, and depression on the relationship between the antecedent factors and excess disability group membership, path analysis was carried out using SEM. All models also included adjustment for age and sex. In the sensitivity analysis, inflammation dyad was also adjusted for (i.e., whether each participant was in the “low inflammation” or the “high inflammation” dyad) (Supplementary Table 1, available on the *Arthritis Care & Research* website at http://onlinelibrary.wiley.com/doi/10.1002/acr.24996). Continuous variables with high variance (age, pain VAS, fatigue VAS, BMI) were standardized. All reported coefficients from the SEM analyses are from fully standardized models to allow direct comparison. The data available in the validation data sets were analyzed using the same strategy.

As 93% of the participants in ESPOIR had no missing data, complete case analysis was performed across all analyses (for a comparison of those included versus excluded, see Supplementary Table 2, available on the *Arthritis Care & Research* website at http://onlinelibrary.wiley.com/doi/10.1002/acr.24996). The confirmatory factor analysis and structural equation models were fit using the lavaan package in R version 3.6.0 (30). The function “modindices” was used to improve the definition of the latent variables until a good model fit was achieved (see above). While there are advantages to performing the mediation analysis within an SEM framework (see above), there is the potential for traditional mediation analysis to be biased beyond simple linear models (31). Therefore, in sensitivity analysis, the mediation analysis was also conducted within a causal mediation framework using the mediation package (32). Other packages used in this analysis were tidyverse, psych, and haven.

## RESULTS

In total, 538 people with RA from the ESPOIR cohort were included in this analysis, of which 200 (37.2%) were in the group characterized by excess disability over the subsequent 10 years. The excess disability group was older at baseline (mean ± SD 50.4 ± 10.7 versus 47.0 ± 12.8 years), had a higher proportion of women (87.0% versus 74.6%) and the same inflammation level (mean ± SD DAS28‐2C score 4.04 ± 1.28 versus 3.99 ± 1.34), as well as higher disability (mean ± SD HAQ score 1.39 ± 0.64 versus 0.93 ± 0.61), more pain, more fatigue, and more anxiety and depression compared with the group with no excess disability (Table 1).

**Table 1:** Baseline demographic and clinical characteristics of individuals in the ESPOIR cohort, stratified by excess disability group status*

Variable	Total ESPOIR cohort	Excess disability	No excess disability	*P*
	(n = 538)	(n = 200)	(n = 338)	
Demographic characteristics				
Age, years	48.3 ± 12.2	50.4 ± 10.7	47.0 ± 12.8	0.0012†
Women, no. (%)	426 (79.2)	174 (87.0)	252 (74.6)	<0.001‡
Symptom duration, months	3.46 ± 1.78	3.63 ± 2.02	3.36 ± 1.62	0.11†
Patient‐reported outcome measures				
Pain VAS (0–100 mm)	40.7 ± 27.5	47.1 ± 27.4	37.0 ± 26.9	<0.001†
Fatigue VAS (0–100 mm)	51.2 ± 27.4	59.3 ± 27.2	46.5 ± 26.5	<0.001†
AIMS anxiety score (0–10)	5.04 ± 2.30	5.61 ± 2.25	4.71 ± 2.27	<0.001†
AIMS depression score (0–10)	3.84 ± 2.13	4.47 ± 2.24	3.47 ± 1.97	<0.001†
HAQ (0–3)	1.10 ± 0.67	1.39 ± 0.64	0.93 ± 0.62	<0.001†
Disease activity				
DAS28 score	4.58 ± 1.15	4.73 ± 1.07	4.48 ± 1.19	0.013†
DAS28 categories, no. (%)				
Remission (DAS28 score <2.6)	25 (4.7)	4 (2.0)	21 (6.2)	0.011‡
Low (DAS28 score ≥2.6 and <3.2)	37 (6.9)	9 (4.5)	28 (8.3)	–
Moderate (DAS28 score ≥3.2 and ≤5.1)	308 (57.2)	120 (60.0)	188 (55.6)	–
High (DAS28 score >5.1)	168 (31.2)	67 (33.5)	101 (29.9)	–
DAS28‐2C score	4.01 ± 1.31	4.04 ± 1.28	3.99 ± 1.34	0.698†
Swollen joint count in 28 joints	7.3 ± 5.4	7.4 ± 5.3	7.3 ± 5.5	0.801†
Tender joint count in 28 joints	9.0 ± 7.2	10.3 ± 7.5	8.3 ± 7.0	0.003†
CRP, mg/liter	22.2 ± 34.0	22.4 ± 32.6	22.1 ± 34.9	0.931†
Patient global VAS (0–100 mm)	62.1 ± 24.5	69.3 ± 22.2	57.9 ± 24.9	<0.001†

* Except where indicated otherwise, values are the mean ± SD. AIMS = arthritis impact measurement scales; CRP = C‐reactive protein; DAS28 = Disease Activity Score in 28 joints; DAS28‐2C = 2‐component Disease Activity Score; ESPOIR = Étude et Suivi des Polyarthrites Indifférenciées Récentes; HAQ = Health Assessment Questionnaire; VAS = visual analog scale.

† By *t*‐test.

‡ By chi‐square test.

### Social support, financial situation, and lifestyle at baseline

Participants in the excess disability group had lower education on average compared with the lower disability group (Table 2). Participants in the excess disability group were less likely to have participated in sport in the previous 6 months prior to baseline (for participation in sport versus no participation in sport, odds ratio [OR] 0.44 [95% confidence interval (95% CI) 0.30, 0.64]), but did not differ in terms of smoking status, alcohol consumption, or BMI (Table 2). In terms of social support, individuals who reported having accommodation support (OR 0.53 [95% CI 0.33, 0.81]), financial support (OR 0.56 [95% CI 0.38, 0.82]), and contact with family (OR 0.55 [95% CI 0.34, 0.90]) were less likely to be in the excess disability group, but being married or cohabiting compared with being single, divorced, or widowed was not associated with excess disability group membership (OR 1.24 [95% CI 0.82, 1.89]) (Table 2).

**Table 2:** Social context, economic factors, education, and lifestyle at baseline, stratified according to excess disability group membership*

Baseline variable	Total ESPOIR cohort	Excess disability	No excess disability	OR of excess disability group membership (95% CI)†
	(n = 538)	(n = 200)	(n = 338)	
Highest educational attainment				
Primary	71 (13.2)	30 (15.0)	41 (12.1)	Ref.
Qualifications at 16 years	190 (35.3)	89 (44.5)	101 (29.9)	1.44 (0.81, 2.57)
Qualifications at 18 years	123 (22.9)	45 (22.5)	78 (23.1)	0.90 (0.48, 1.70)
Undergraduate	83 (15.4)	17 (8.5)	66 (19.5)	0.44 (0.20, 0.92)
Postgraduate	71 (13.2)	19 (9.5)	52 (15.4)	0.60 (0.28, 1.24)
Lifestyle				
Current smoker	265 (49.3)	100 (50.0)	165 (48.8)	1.35 (0.93, 1.96) [Ref. not smoking]
Alcohol consumption	102 (19.0)	32 (16.0)	70 (20.7)	0.77 (0.47, 1.24) [Ref. no consumption]
Participation in sport in the previous 6 months	213 (39.6)	55 (27.5)	158 (46.7)	0.44 (0.30, 0.64) [Ref. no sport]
BMI, mean ± SD	25.6 ± 4.8	26.1 ± 5.1	25.3 ± 4.6	1.03 (0.99, 1.07)‡
Social support				
Accommodation support available	434 (80.7)	147 (73.5)	287 (84.9)	0.52 (0.33, 0.81) [Ref. no support]
Financial support available	385 (71.6)	127 (63.5)	258 (76.3)	0.56 (0.38, 0.82) [Ref. no support]
Contact with family	455 (84.6)	158 (79.0)	297 (87.9)	0.55 (0.34, 0.90) [Ref. no contact]
Married/cohabiting	396 (73.6)	151 (75.5)	245 (72.5)	1.24 (0.82, 1.89) [Ref. single, divorced or widowed]
Personal economic situation				
Family income				
<€610	22 (4.1)	11 (5.5)	11 (3.3)	2.29 (0.89, 5.91)
€610–1,220	94 (17.5)	41 (20.5)	53 (15.7)	1.51 (0.87, 2.60)
€1,220–1,830	119 (22.1)	50 (25.0)	69 (20.4)	1.57 (0.94, 2.64)
€1,830–2,440	108 (20.1)	32 (16.0)	76 (22.5)	0.88 (0.51, 1.52)
€2,440–2,745	48 (8.9)	17 (8.5)	31 (9.2)	1.19 (0.58, 2.38)
>€2,745	147 (27.3)	49 (24.5)	98 (29.0)	Ref.
Personal income				
<€610	110 (20.4)	52 (26.0)	58 (17.2)	4.10 (1.51, 13.18)
€610–1,220	185 (34.4)	72 (36.0)	113 (33.4)	3.12 (1.20, 9.76)
€1220–1,830	138 (25.7)	48 (24.0)	90 (26.6)	2.77 (1.05, 8.73)
€1830–2,440	65 (12.1)	21 (10.5)	44 (13.0)	2.41 (0.84, 8.04)
€2440–2,745	10 (1.9)	2 (1.0)	8 (2.4)	1.28 (0.16, 7.55)
>€2,745	30 (5.6)	5 (2.5)	25 (7.4)	Ref.
Homeowner	329 (61.2)	120 (60.0)	209 (61.8)	0.77 (0.52, 1.13) [Ref. not homeowner]
Show/cinema visit	322 (59.9)	101 (50.5)	221 (65.4)	0.53 (0.37, 0.77) [Ref. no show/cinema visits]
Holiday	293 (54.5)	90 (45.0)	203 (60.1)	0.50 (0.35, 0.72) [Ref. no holidays]
Job status				
Working (full‐time/part‐time)				
Not working§	319 (59.3)	107 (53.5)	212 (62.7)	Ref.
Retired	118 (21.9)	54 (27.0)	64 (18.9)	1.49 (0.95, 2.33)
Job level	101 (18.8)	39 (19.5)	62 (18.3)	0.64 (0.36, 1.14)
Low	134 (24.9)	57 (28.5)	77 (22.8)	1.40 (0.91, 2.16)
Medium	348 (64.7)	134 (67.0)	214 (63.3)	Ref.
High	56 (10.4)	9 (4.5)	47 (13.9)	0.33 (0.15, 0.69)
Rural/urban dwelling				
Population of participant's town				
<5,000 inhabitants	188 (34.9)	76 (38.0)	112 (33.1)	Ref.
5,000–20,000 inhabitants	95 (17.7)	39 (19.5)	56 (16.6)	1.08 (0.64, 1.81)
20,000–50,000 inhabitants	109 (20.3)	35 (17.5)	74 (21.9)	0.69 (0.41, 1.14)
>50,000 inhabitants	146 (27.1)	50 (25.0)	96 (28.4)	0.79 (0.50, 1.25)
Life events				
Traumatic event in the previous 6 months	59 (11.0)	29 (14.5)	30 (8.8)	1.51 (0.86, 2.65) [Ref. no event]
Death of someone close in the previous 6 months	64 (11.9)	30 (15.0)	34 (10.1)	1.47 (0.86, 2.51) [Ref. no event]

* Except where indicated otherwise, values are the number (%) of participants. 95% CI = 95% confidence interval; BMI = body mass index; ESPOIR = Étude et Suivi des Polyarthrites Indifférenciées Récentes; OR = odds ratio; Ref. = reference category.

† Odds ratios (ORs) from logistic regression. Each factor was tested in separate models (rather than a single multivariable model) and was adjusted for age and sex.

‡ Analyzed as a continuous scale.

§ At home, unemployed, student, disabled, and long‐term illness.

Regarding an individual's personal financial situation at baseline, not working (at home, unemployed, student, disabled, long‐term illness) was associated with higher odds of being in the excess disability group compared with working full‐ or part‐time (OR 1.49 [95% CI 0.95, 2.33]). Baseline higher job level was associated with lower odds of being in the excess disability group (low versus medium, OR 1.40 [95% CI 0.91, 2.16]; high versus medium, OR 0.33 [0.15, 0.69]). Baseline higher personal and family income was also associated with lower odds of being in the excess disability group, as was being a homeowner (OR 0.77 [95% CI 0.52, 1.13]), being able to go to a show or the cinema (OR 0.53 [95% CI 0.37, 0.77]), and being able to go on holiday at baseline (OR 0.50 [95% CI 0.35, 0.72]) (Table 2). Last, a traumatic event or the death of someone close in the 6 months preceding baseline were associated with 50% increased odds of being in the excess disability group, as was living in a rural as opposed to an urban environment (although the CIs overlapped 1) (Table 2).

### Definition of latent variables

**Figure 1:**
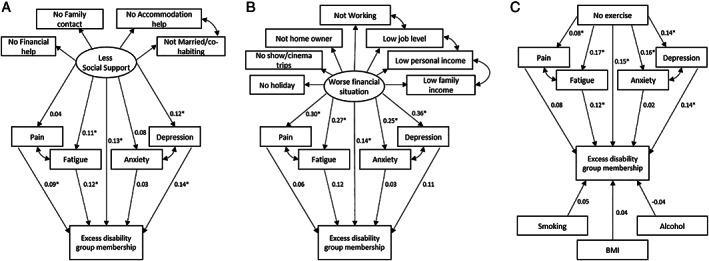
Structural equation modeling (SEM) diagrams of direct and mediating paths between baseline latent variables less social support (**A**), worse financial situation (**B**), as well as lifestyle factors and excess disability group membership (**C**). All models were also adjusted for age and sex. Ovals represent latent constructs, and rectangles represent observed variables. The total effects referred to in the rest of the paper combine both the direct effect from the latent constructs to the outcome (excess disability), and the indirect effects through the patient‐reported outcome measures. For example, the total effect of social support on excess disability (**A**) was 0.17, calculated as the direct effect (0.13) plus the indirect effects through the patient‐reported outcome measures ([0.04 × 0.09] + [0.11 × 0.12] + [0.08 × 0.03] + [0.12 × 0.14]). The proportion mediated by the patient‐reported outcome measures is the indirect effect divided by the total effect. BMI = body mass index. * = statistically significant.

Many of the social support, financial, and lifestyle variables are correlated. Therefore, latent variables were constructed to summarize these correlated variables (Figure 1). Confirmatory factor analysis indicated good model fit, supporting these latent variables fit the data (for social support, TLI = 0.996 and RMSEA = 0.020; for financial situation, TLI = 0.892 and RMSEA = 0.054). Sport, smoking, alcohol consumption, and BMI were analyzed as individual components within a SEM, since no latent variables combining these indicators had satisfactory model fit (Figure 1C).

### Relationship between latent variables and excess disability group membership

Less social support (β = 0.17 [95% CI 0.08, 0.26]), worse financial situation (β = 0.24 [95% CI 0.14, 0.34]), less participation in sport in the previous 6 months (β = 0.17 [95% CI 0.09, 0.25]), and less education (β = 0.15 [95% CI 0.06, 0.23]) were all associated with excess disability group membership (Table 3 and Figure 1). However, smoking (β = 0.05 [95% CI –0.03, 0.14]), alcohol consumption (β = –0.04 [95% CI –0.12, 0.04]), and BMI (β = 0.04 [95% CI –0.05, 0.12]) were not associated with excess disability group membership.

**Table 3:** Results from SEMs testing the relationships between latent variables, exercise, and education with excess disability*

Mediation SEM	Social support	Financial status	Exercise	Education
Total effect on high HAQ group membership	0.168 (0.076, 0.260)	0.237 (0.138, 0.335)	0.173 (0.093, 0.254)	0.149 (0.064, 0.233)
Direct effect	0.132 (0.041, 0.223)	0.141 (0.025, 0.257)	0.124 (0.043, 0.205)	0.082 (–0.004, 0.168)
Proportion of total effect unexplained by patient‐reported outcome measures	79 (41, 98)	59 (18, 82)	71 (42, 87)	55 (2, 77)
Indirect effect through pain	0.003 (–0.006, 0.012)	0.017 (–0.010, 0.044)	0.006 (–0.003, 0.016)	0.015 (–0.004, 0.035)
Proportion mediated through pain	2 (–6, 10)	7 (–4, 23)	3 (–1, 12)	10 (–2, 33)
Indirect effect through fatigue	0.014 (–0.001, 0.028)	0.032 (0.006, 0.057)	0.020 (0.002, 0.038)	0.022 (0.004, 0.041)
Proportion mediated through fatigue	8 (0, 25)	13 (3, 30)	12 (2, 29)	15 (4, 39)
Indirect effect through depression	0.017 (–0.001, 0.034)	0.041 (0.003, 0.078)	0.020 (0.002, 0.039)	0.025 (0.004, 0.047)
Proportion mediated through depression	10 (0, 32)	17 (0, 43)	12 (2, 28)	17 (3, 45)
Indirect effect through anxiety	0.002 (–0.006, 0.011)	0.006 (–0.018, 0.031)	0.003 (–0.012, 0.019)	0.003 (–0.008, 0.015)
Proportion mediated through anxiety	1 (–5, 10)	3 (–9, 16)	2 (–7, 14)	2 (–5, 15)

* Values are the standardized β value or percentage (95% confidence interval). Analyses were also adjusted for age and gender. HAQ = Health Assessment Questionnaire; SEM = structural equation modeling.

**Figure 2:**
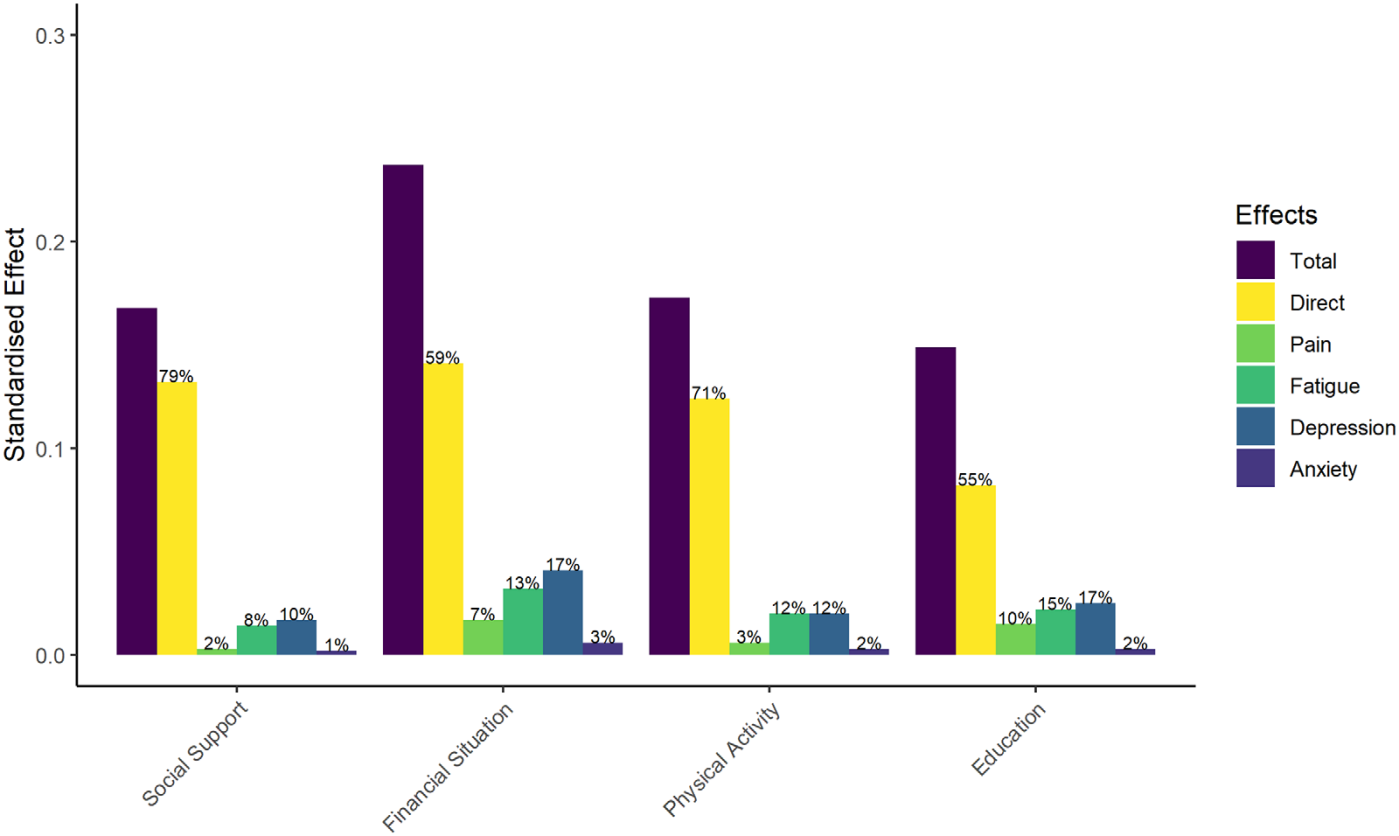
Bar chart illustrating the total effect of each antecedent factor on excess disability and how these total effects are broken down into direct and indirect effects. Percentages on the top of the bars represent the proportion of each direct and indirect effect on the total effect—e.g., the total effect of social support is made up of 79% “direct effect,” and 2% of the effect is mediated by pain, 8% by fatigue, 10% by depression, and 1% by anxiety.

Regarding the mediating effect of the patient‐reported outcome measures, pain and anxiety did not mediate the effect of any of the social, economic, or lifestyle factors (Table 3). However, fatigue and depression each mediated between 10% and 17% of the effect of these factors (Table 3 and Figure 2). These findings were confirmed using a causal mediation analysis framework (Supplementary Tables 3 and 4, available on the *Arthritis Care & Research* website at http://onlinelibrary.wiley.com/doi/10.1002/acr.24996).

### Validation analyses—NOAR and ERAN

In total, 416 people had complete data and were included in the NOAR analysis (excess disability = 166 [39.9%], lower disability = 250 [60.1%]) (baseline demographic and clinical characteristics are shown in Supplementary Table 5, available at http://onlinelibrary.wiley.com/doi/10.1002/acr.24996). Not working and having a lower job status at baseline were associated with increased odds of being in the excess disability group (Supplementary Table 6, available at http://onlinelibrary.wiley.com/doi/10.1002/acr.24996). The total effect of working status was β 0.18 (95% CI 0.07, 0.29, adjusted for age and sex), of which 49% was a direct effect, with the remaining effect mediated by pain, fatigue, and depression (Supplementary Table 7, available at http://onlinelibrary.wiley.com/doi/10.1002/acr.24996). A similar relationship was observed for job status (Supplementary Table 7).

The ERAN analysis included 386 individuals (excess disability = 198 [51.3%], lower disability = 188 [48.7%]) (for baseline demographic and clinical characteristics, see Supplementary Table 5, available at http://onlinelibrary.wiley.com/doi/10.1002/acr.24996). In ERAN, higher deprivation was weakly associated with increased odds of being in the high HAQ group, and the CI contained the null (OR per decile increase in deprivation [IMD] 1.06 [95% CI 0.97, 1.15]) (Supplementary Table 6, available at http://onlinelibrary.wiley.com/doi/10.1002/acr.24996). Since the effect of IMD was weak and the sample size was small, the CIs from the path analysis were wide (Supplementary Table 7).

## DISCUSSION

This analysis of a large cohort of individuals with RA illustrates the importance of social and financial factors and lifestyle behaviors in influencing excess disability occurring in RA, independent of inflammation level. Individuals who had excess disability over 10 years with respect to their inflammation levels were more likely to have less social support, poorer financial status (less disposable income, less likely to be homeowners, working in lower‐level occupations), lower education, and exercise less at baseline. Previous research highlighted the role pain, fatigue, and depression may play in driving excess disability in RA (8). This analysis illustrates that, while some of the effect of the aforementioned antecedent social and financial factors was mediated by fatigue and depression in the ESPOIR cohort, a significant proportion was not explained by these patient‐reported outcome measures. Therefore, social inequality is potentially an important factor influencing long‐term disability in RA, alongside inflammation, pain, fatigue, and depression. Addressing the clear social inequality in RA outcomes should be more prominently addressed in RA management strategies and guidelines.

Social and economic factors have been shown to correlate with RA outcomes in previous studies. Cross‐sectional and short‐term follow‐up studies have reported associations between social support and depression (15) and psychological distress (17), as well as relationships between income and disability (33, 34, 35, 36). Furthermore, physical activity is an established intervention that improves disability in RA (12, 37, 38), and people with early RA and lower socioeconomic status are less likely to perform physical activity in the early phases of RA (39). The current analysis extends these cross‐sectional and short‐term follow‐up studies to show that social support, financial factors, and exercise prior to RA onset predict outcome trajectories over 10 years following symptom onset.

Social support is a potentially vital resource for dealing with a wide variety of stressors, such as RA and the disability that may follow (the “buffering” hypothesis [40]) (41), whereas social isolation is associated with poor health and greater risk of death (2). This social support may influence disability in 2 ways: 1) health‐facilitating function (e.g., encouragement, motivation), and 2) stress‐reducing function (e.g., facilitating cognitive and practical adjustment) (42). RA can also have significant economic implications (43), and individuals with higher disability earn even less in the years after diagnosis (44). Potentially greater economic reserves mean patients are better able to adapt to RA onset and thus experience lower disability (45) as well as potentially being able to access advanced therapies in certain health care settings (46, 47), or perhaps higher economic level and more education and health literacy allow people with RA greater autonomy in terms of positive health behaviors and seeking support (48). Therefore, rheumatology teams may need to identify individuals with these characteristics for enhanced follow‐up, potentially through digital modalities or referral to additional nonpharmacologic interventions (e.g., physical activity, psychological, or self‐management interventions [49, 50]). Signposting to patient organizations may also be beneficial in order to tackle social isolation. Furthermore, greater macro‐level changes need to be implemented to reduce the social gradients of RA outcomes observed in these analyses.

This study has several strengths. The ESPOIR cohort is large, and the multicenter design means the population is representative of French regional variation, with extensive data on social and economic factors. Furthermore, while identical analyses could not be performed in NOAR and ERAN due to differences in available data regarding antecedent social and economic factors, a similar interpretation of the results from analyses of these data sets was made (i.e., that antecedent economic factors are associated with excess disability in RA), in part substantiating the generalizability of the findings. The reported level of alcohol consumption was low, potentially indicating social desirability bias. The use of “traditional” mediation analysis can be biased in situations with nonlinear effects (31). However, a sensitivity analysis using a causal mediation approach demonstrated similar results, indicating minimal bias. Attempts to include all the exposure variables within a single SEM were unsuccessful due to problems with model convergence (perhaps due to limitations in statistical power). Since there could potentially be some correlation between the latent variables in this analysis, a hypothetical model that included all the antecedent variables within this paper may provide attenuated effect estimates of the associations between each factor and excess disability, compared with the separate models within this paper. Future analyses with larger sample sizes should aim to combine all these antecedent factors into single models to separate out the individual effects. There was a higher proportion of missing data in the validation data sets compared with ESPOIR. While a sensitivity analysis from a previous analysis showed minimal bias from these missing data (8), the validation analyses of the current paper may be susceptible to some missing data bias.

In conclusion, social support, personal financial situation, education, and exercise were associated with membership in groups characterized by excess disability over 10 years following the onset of symptoms. These effects were largely independent of baseline patient‐reported outcome measures. This indicates the pivotal importance social and economic factors play in explaining the inflammation–disability gap evident in long‐term outcomes in individuals with RA, and these factors require greater prominence in RA management strategies and guidelines.

## AUTHOR CONTRIBUTIONS

All authors were involved in drafting the article or revising it critically for important intellectual content, and all authors approved the final version to be published. Dr. Gwinnutt had full access to all of the data in the study and takes responsibility for the integrity of the data and the accuracy of the data analysis.

### Study conception and design

Gwinnutt, Norton, Hyrich, Verstappen.

### Acquisition of data

Combe, Rincheval, Ruyssen‐Witrand, Fautrel, McWilliams, Walsh, Nikiphorou, Kiely, Young, Chipping, MacGregor, Verstappen.

### Analysis and interpretation of data

Gwinnutt, Norton, Hyrich, Lunt, Verstappen.

## Supporting information


Disclosure Form

**Appendix S1.** Supplementary Information

## References

[1] Lewer D , Jayatunga W , Aldridge RW , et al. Premature mortality attributable to socioeconomic inequality in England between 2003 and 2018: an observational study. Lancet Public Health 2020;5:e33–41. 31813773 10.1016/S2468-2667(19)30219-1 PMC7098478

[2] Holt‐Lunstad J , Smith TB , Layton JB . Social relationships and mortality risk: a meta‐analytic review. PLoS Med 2010;7:e1000316. 20668659 10.1371/journal.pmed.1000316 PMC2910600

[3] Palmer RC , Ismond D , Rodriquez EJ , et al. Social determinants of health: future directions for health disparities research. Am J Public Health 2019;109:S70–1. 30699027 10.2105/AJPH.2019.304964 PMC6356128

[4] Smolen JS , Aletaha D , Barton A , et al. Rheumatoid arthritis. Nat Rev Dis Primers 2018;4:18001. 29417936 10.1038/nrdp.2018.1

[5] Gwinnutt JM , Symmons DP , Macgregor AJ , et al. Twenty‐year outcome and association between early treatment and mortality and disability in an inception cohort of patients with rheumatoid arthritis: results from the Norfolk Arthritis Register. Arthritis Rheumatol 2017;69:1566–75. 28425173 10.1002/art.40090 PMC5600136

[6] Gwinnutt JM , Symmons DP , Macgregor AJ , et al. Have the 10‐year outcomes of patients with early inflammatory arthritis improved in the new millennium compared with the decade before? Results from the Norfolk Arthritis Register. Ann Rheum Dis 2018;77:848–54. 29475855 10.1136/annrheumdis-2017-212426 PMC5965352

[7] Kapetanovic MC , Lindqvist E , Nilsson JA , et al. Development of functional impairment and disability in rheumatoid arthritis patients followed for 20 years: relation to disease activity, joint damage, and comorbidity. Arthritis Care Res (Hoboken) 2015;67:340–8. 25186552 10.1002/acr.22458

[8] Gwinnutt JM , Norton S , Hyrich K , et al. Exploring the disparity between inflammation and disability in the 10‐year outcomes of people with rheumatoid arthritis. Rheumatology (Oxford) 2022;keac137. 10.1093/rheumatology/keac137 PMC9707289 35274696

[9] Norton S , Fu B , Scott DL , et al. Health Assessment Questionnaire disability progression in early rheumatoid arthritis: systematic review and analysis of two inception cohorts. Semin Arthritis Rheum 2014;44:131–44. 24925692 10.1016/j.semarthrit.2014.05.003 PMC4282305

[10] Molina E , del Rincon I , Restrepo JF , et al. Association of socioeconomic status with treatment delays, disease activity, joint damage, and disability in rheumatoid arthritis. Arthritis Care Res (Hoboken) 2015;67:940–6. 25581770 10.1002/acr.22542 PMC4482767

[11] Kiadaliri AA , Petersson IF , Englund M . Educational inequalities in mortality associated with rheumatoid arthritis and other musculoskeletal disorders in Sweden. BMC Musculoskelet Disord 2019;20:83. 30777043 10.1186/s12891-019-2465-8 PMC6379941

[12] Gwinnutt JM , Verstappen SM , Humphreys JH . The impact of lifestyle behaviours, physical activity and smoking on morbidity and mortality in patients with rheumatoid arthritis. Best Pract Res Clin Rheumatol 2020;34:101562. 32646673 10.1016/j.berh.2020.101562

[13] Gwinnutt JM , Wieczorek M , Cavalli G , et al. Effects of physical exercise and body weight on disease‐specific outcomes of people with rheumatic and musculoskeletal diseases (RMDs): systematic reviews and meta‐analyses informing the 2021 EULAR recommendations for lifestyle improvements in people with RMDs. RMD Open 2022;8:e002168. 35361692 10.1136/rmdopen-2021-002168 PMC8971792

[14] Wieczorek M , Gwinnutt JM , Ransay M , et al. Smoking, alcohol consumption and disease‐specific outcomes in rheumatic and musculoskeletal diseases (RMDs): systematic reviews informing the 2021 EULAR recommendations for lifestyle improvements in people with RMDs. RMD Open 2022;8:e002170. 35351808 10.1136/rmdopen-2021-002170 PMC8966569

[15] Brandstetter S , Riedelbeck G , Steinmann M , et al. Pain, social support and depressive symptoms in patients with rheumatoid arthritis: testing the stress‐buffering hypothesis. Rheumatol Int 2017;37:931–6. 28124095 10.1007/s00296-017-3651-3

[16] Strating MM , Suurmeijer TP , van Schuur WH . Disability, social support, and distress in rheumatoid arthritis: results from a thirteen‐year prospective study. Arthritis Rheum 2006;55:736–44. 17013871 10.1002/art.22231

[17] Benka J , Nagyova I , Rosenberger J , et al. Social support and psychological distress in rheumatoid arthritis: a 4‐year prospective study. Disabil Rehabil 2012;34:754–61. 22004369 10.3109/09638288.2011.619618

[18] Lin KC , Chen PC , Twisk JW , et al. Time‐varying nature of risk factors for the longitudinal development of disability in older adults with arthritis. J Epidemiol 2010;20:460–7. 20838022 10.2188/jea.JE20090154 PMC3900823

[19] Combe B , Benessiano J , Berenbaum F , et al. The ESPOIR cohort: a ten‐year follow‐up of early arthritis in France: methodology and baseline characteristics of the 813 included patients. Joint Bone Spine 2007;74:440–5. 17905631 10.1016/j.jbspin.2007.06.001

[20] Sass C , Moulin JJ , Guéguen R . Le score epices: un score individuel de précarité. Construction du score et mesure des relations avec des données de santé, dans une population de 197 389 personnes. Bull Epidemiol Hebd 2006;14:93–6.

[21] Labbe E , Blanquet M , Gerbaud L , et al. A new reliable index to measure individual deprivation: the EPICES score. Eur J Public Health 2015;25:604–9. 25624273 10.1093/eurpub/cku231

[22] Guillemin F , Braincon S , Pourel J . Measurement of the functional capacity in rheumatoid polyarthritis: a French adaptation of the Health Assessment Questionnaire (HAQ). Rev Rhum Mal Osteoartic 1991;58:459–65. In French. 1896787

[23] Pouchot J , Guillemin F , Coste J , et al. Validity, reliability, and sensitivity to change of a French version of the arthritis impact measurement scales 2 (AIMS2) in patients with rheumatoid arthritis treated with methotrexate. J Rheumatol 1996;23:52–60. 8838508

[24] Fransen J , Welsing P , de Keijzer R , et al. Disease activity scores using C‐reactive protein: CRP may replace ESR in the assessment of RA disease activity [abstract]. Ann Rheum Dis 2004;62 Suppl 1:151.

[25] Hensor EM , McKeigue P , Ling SF , et al. Validity of a two‐component imaging‐derived disease activity score for improved assessment of synovitis in early rheumatoid arthritis. Rheumatology (Oxford) 2019;58:1400–9. 30824919 10.1093/rheumatology/kez049 PMC6649844

[26] Kirwan JR , Reeback JS . Stanford Health Assessment Questionnaire modified to assess disability in British patients with rheumatoid arthritis. Br J Rheumatol 1986;25:206–9. 3708236 10.1093/rheumatology/25.2.206

[27] Symmons DP , Barrett EM , Bankhead CR , et al. The incidence of rheumatoid arthritis in the United Kingdom: results from the Norfolk Arthritis Register. Br J Rheumatol 1994;33:735–9. 8055200 10.1093/rheumatology/33.8.735

[28] Garwood W. The Early Rheumatoid Arthritis Network (ERAN). Musculoskeletal Care 2004;2:240–4. 17041987 10.1002/msc.75

[29] Ministry of Housing, Communities and Local Government . Index of multiple deprivation score, 2007 2015. URL: https://data.gov.uk/dataset/5ceb7e93‐bc1a‐48cf‐80fd‐fbdd15909640/index‐of‐multiple‐deprivation‐score‐2007.

[30] Rosseel Y. lavaan: an R package for structural equation modeling. J Stat Software 2012;48:1–36.

[31] Lange T , Vansteelandt S , Bekaert M . A simple unified approach for estimating natural direct and indirect effects. Am J Epidemiol 2012;176:190–5. 22781427 10.1093/aje/kwr525

[32] Tingley D , Yamamoto T , Hirose K , et al. mediation: R Package for causal mediation analysis. J Stat Software 2014;59:1–38.

[33] Zhao S , Chen Y , Chen H . Sociodemographic factors associated with functional disability in outpatients with rheumatoid arthritis in Southwest China. Clin Rheumatol 2015;34:845–51. 25687985 10.1007/s10067-015-2896-z

[34] Alarcón AM , Muñoz S , Kaufman JS , et al. Contribution of ethnic group and socioeconomic status to degree of disability in rheumatoid arthritis in Chilean patients. Rheumatol Int 2015;35:685–9. 25178741 10.1007/s00296-014-3123-y

[35] Callhoff J , Luque Ramos A , Zink A , et al. The association of low income with functional status and disease burden in German patients with rheumatoid arthritis: results of a cross‐sectional questionnaire survey based on claims data. J Rheumatol 2017;44:766–72. 28412709 10.3899/jrheum.160966

[36] Yang G , Bykerk VP , Boire G , et al. Does socioeconomic status affect outcomes in early inflammatory arthritis? Data from a Canadian multisite suspected rheumatoid arthritis inception cohort. J Rheumatol 2015;42:46–54. 25399388 10.3899/jrheum.131382

[37] Baillet A , Vaillant M , Guinot M , et al. Efficacy of resistance exercises in rheumatoid arthritis: meta‐analysis of randomized controlled trials. Rheumatology (Oxford) 2012;51:519–27. 22120463 10.1093/rheumatology/ker330

[38] Baillet A , Zeboulon N , Gossec L , et al. Efficacy of cardiorespiratory aerobic exercise in rheumatoid arthritis: meta‐analysis of randomized controlled trials. Arthritis Care Res (Hoboken) 2010;62:984–92. 20589690 10.1002/acr.20146

[39] Gwinnutt JM , Alsafar H , Hyrich KL , et al. Do people with rheumatoid arthritis maintain their physical activity level at treatment onset over the first year of methotrexate therapy? Rheumatology (Oxford) 2021;60:4633–42. 33605404 10.1093/rheumatology/keab060 PMC8487269

[40] Cohen S , Wills TA . Stress, social support, and the buffering hypothesis. Psychol Bull 1985;98:310–57. 3901065

[41] Tough H , Siegrist J , Fekete C . Social relationships, mental health and wellbeing in physical disability: a systematic review. BMC Public Health 2017;17:414. 28482878 10.1186/s12889-017-4308-6 PMC5422915

[42] Krol B , Sanderman R , Suurmeijer TP . Social support, rheumatoid arthritis and quality of life: concepts, measurement and research. Patient Educ Couns 1993;20:101–20. 8337188 10.1016/0738-3991(93)90125-g

[43] Furneri G , Mantovani LG , Belisari A , et al. Systematic literature review on economic implications and pharmacoeconomic issues of rheumatoid arthritis. Clin Exp Rheumatol 2012;30 Suppl 73:S72–84. 23072761

[44] Wolfe F , Michaud K , Choi HK , et al. Household income and earnings losses among 6,396 persons with rheumatoid arthritis. J Rheumatol 2005;32:1875–83. 16206340

[45] Rojas M , Rodriguez Y , Pacheco Y , et al. Resilience in women with autoimmune rheumatic diseases. Joint Bone Spine 2018;85:715–20. 29289647 10.1016/j.jbspin.2017.12.012

[46] DeWitt EM , Lin L , Glick HA , et al. Pattern and predictors of the initiation of biologic agents for the treatment of rheumatoid arthritis in the United States: an analysis using a large observational data bank. Clin Ther 2009;31:1871–80; discussion 1858. 19808146 10.1016/j.clinthera.2009.08.020 PMC3518838

[47] Yelin E , Tonner C , Kim SC , et al. Sociodemographic, disease, health system, and contextual factors affecting the initiation of biologic agents in rheumatoid arthritis: a longitudinal study. Arthritis Care Res (Hoboken) 2014;66:980–9. 24339352 10.1002/acr.22244 PMC4124524

[48] Caplan L , Wolfe F , Michaud K , et al. Strong association of health literacy with functional status among rheumatoid arthritis patients: a cross‐sectional study. Arthritis Care Res (Hoboken) 2014;66:508–14. 24023051 10.1002/acr.22165

[49] Nagy G , Roodenrijs NM , Welsing PM , et al. EULAR points to consider for the management of difficult‐to‐treat rheumatoid arthritis. Ann Rheum Dis 2022;81:20–33. 34407926 10.1136/annrheumdis-2021-220973 PMC8761998

[50] Nikiphorou E , Santos EJ , Marques A , et al. 2021 EULAR recommendations for the implementation of self‐management strategies in patients with inflammatory arthritis. Ann Rheum Dis 2021;80:1278–85. 33962964 10.1136/annrheumdis-2021-220249 PMC8458093

