# Supplementary material for: Influence of Social Support, Financial Status, and Lifestyle on the Disparity Between Inflammation and Disability in Rheumatoid Arthritis
Source: Arthritis Care Res (Hoboken). 2022 Dec 28;75(5):1026–35. doi: 10.1002/acr.24996 (PMC10952173; doi:10.1002/acr.24996)
Supplement: Supplementary file 2 — Appendix S1. Supplementary Information [file ACR-75-1026-s001.docx]

Supplementary files


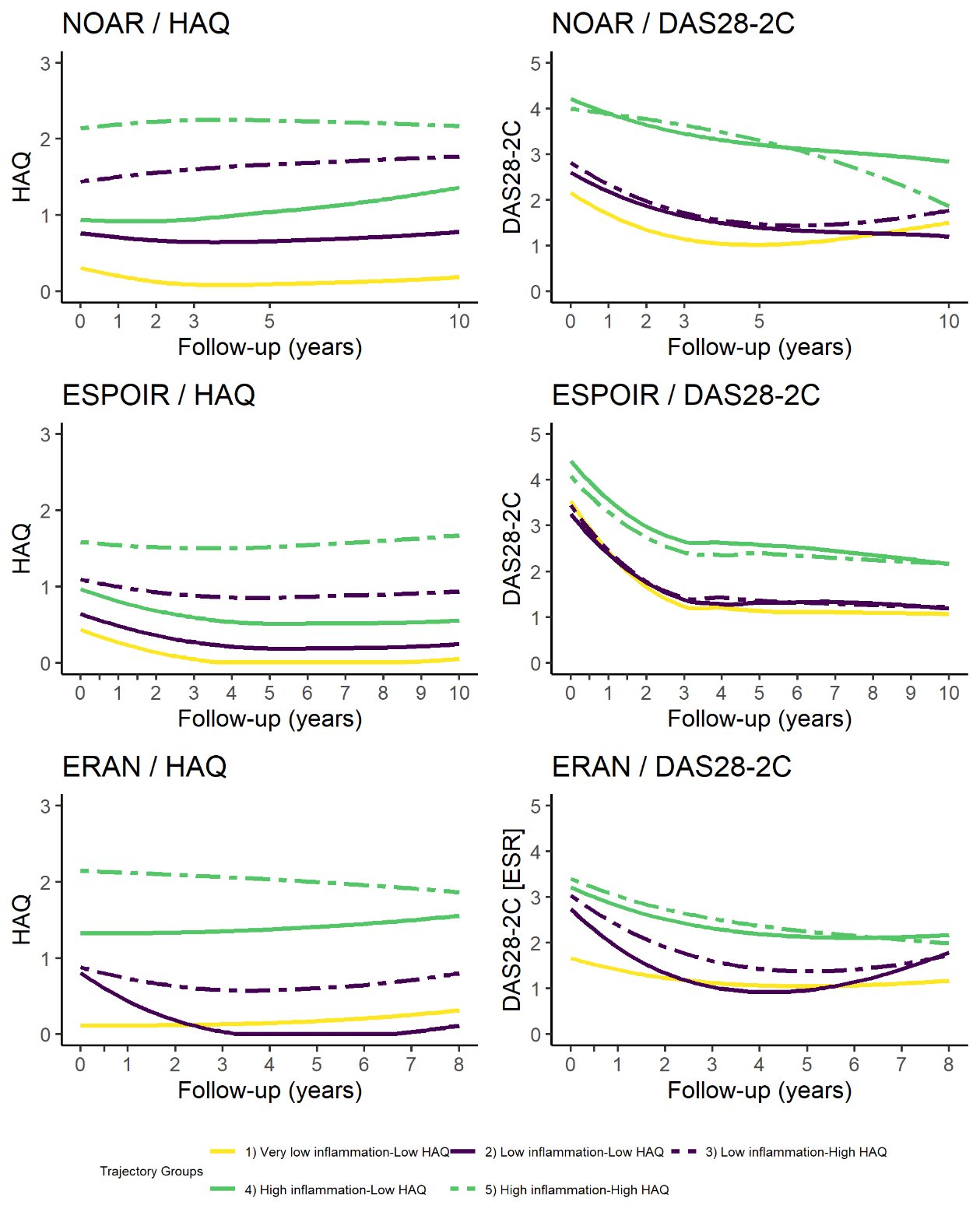
Title: The influence of social support, financial context and lifestyle on the disparity between inflammation and disability in rheumatoid arthritis

Supplementary Figure 1 – Trajectories of inflammation and disability across the three cohorts identified in a previous analysis (Gwinnutt et al, 2022, Exploring the disparity between inflammation and disability in the 10-year outcomes of people with rheumatoid arthritis, Rheumatology (Oxford), Online ahead of print, keac137. doi: 10.1093/rheumatology/keac137 ^1^)

| *Supplementary Table 1 – Results from structural equation models with additional adjustment for inflammation dyad group* | | | | |
| --- | --- | --- | --- | --- |
|  | **Social Support** | **Financial Status** | **Exercise** | **Education** |
| Mediation SEM | **Standardised β (95% CI)** | **Standardised β (95% CI)** | **Standardised β (95% CI)** | **Standardised β (95% CI)** |
| Total Effect on high HAQ membership | 0.166 (0.074, 0.258) | 0.243 (0.140, 0.345) | 0.173 (0.092, 0.253) | 0.142 (0.058, 0.226) |
| Direct effect | 0.133 (0.043, 0.224) | 0.154 (0.039, 0.270) | 0.127 (0.046, 0.208) | 0.082 (-0.003, 0.168) |
| Proportion of total effect unexplained by PROMs | 80% (45%, 100%) | 64% (29%, 84%) | 74% (46%, 88%) | 58% (2%, 79%) |
| Indirect effect through pain | 0.002 (-0.006, 0.011) | 0.016 (-0.010, 0.041) | 0.005 (-0.004, 0.014) | 0.015 (-0.005, 0.034) |
| Proportion mediated through pain | 1% (-6%, 9%) | 6% (4%, 21%) | 3% (-1%, 12%) | 10% (-2%, 34%) |
| Indirect effect through fatigue | 0.013 (-0.002, 0.027) | 0.028 (0.006, 0.051) | 0.019 (0.002, 0.036) | 0.020 (0.003, 0.038) |
| Proportion mediated through fatigue | 8% (1%, 23%) | 12% (3%, 27%) | 11% (2%, 27%) | 14% (3%, 38%) |
| Indirect effect through depression | 0.016 (-0.001, 0.033) | 0.039 (0.006, 0.072) | 0.019 (0.001, 0.036) | 0.022 (0.003, 0.041) |
| Proportion mediated through depression | 9% (-1%, 32%) | 16% (2%, 38%) | 11% (2%, 26%) | 16% (2%, 42%) |
| Indirect effect through anxiety | 0.002 (-0.005, 0.009) | 0.005 (-0.015, 0.026) | 0.003 (-0.011, 0.017) | 0.003 (-0.007, 0.013) |
| Proportion mediated through anxiety | 1% (-5%, 9%) | 2% (-7%, 13%) | 2% (-6%, 12%) | 2% (-4%, 14%) |
| *Analyses also adjusted for age, gender and inflammation severity pair* | | | | |

| *Supplementary table 2 – Comparing participants included in the ESPOIR analysis to the participants removed due to missing data* | | | | |
| --- | --- | --- | --- | --- |
| Variable | **ESPOIR cohort included in analysis,**  **Mean (SD) / N (%)** | | **Participants excluded,**  **Mean (SD) / N (%)** | **p** |
| N | | 538 | 42 |  |
| *Demographics* | |  |  |  |
| Age, years | | 48.3 (12.2) | 52.3 (12.4) | 0.001† |
| Women, N(%) | | 426 (79.2%) | 34 (81.0%) | 0.940‡ |
| Symptom duration, months | | 3.46 (1.78) | 3.66 (1.37) | 0.384 |
| *PROMs* | |  |  |  |
| Pain VAS (0-100) | | 40.7 (27.5) | 36.2 (28.4) | 0.329† |
| Fatigue VAS (0-100) | | 51.2 (27.4) | 44.3 (28.6) | 0.139† |
| AIMS anxiety (0-10) | | 5.04 (2.30) | 5.07 (2.92) | 0.958† |
| AIMS depression (0-10) | | 3.84 (2.13) | 3.46 (2.26) | 0.305† |
| HAQ (0-3) | | 1.10 (0.67) | 1.03 (0.75) | 0.580† |
| *Disease activity* | |  |  |  |
| DAS28 | | 4.58 (1.15) | 4.49 (1.08) | 0.622† |
| DAS28-2C | | 4.01 (1.31) | 4.18 (1.17) | 0.381† |
| Swollen joint count 28 | | 7.3 (5.4) | 8.12 (5.2) | 0.362† |
| Tender joint count 28 | | 9.0 (7.2) | 7.7 (5.9) | 0.156† |
| CRP, mg/l | | 22.2 (34.0) | 24.9 (40.8) | 0.681† |
| Patient global VAS (0-100) | | 62.1 (24.5) | 60.6 (27.6) | 0.741† |
| *† t-test. ‡ chi^2^ test*  *AIMS = Arthritis Impact Measurement Scales, CRP = C-reactive protein, DAS28 = Disease Activity Score 28, DAS28-2C = two-component Disease Activity Score, ESPOIR = Étude et Suivi des Polyarthrites Indifférenciées Récentes, HAQ = Health Assessment Questionnaire, N = number, PROMS = patient reported outcome measures, SD = standard deviation, VAS = visual analogue scale* | | | | |

| *Supplementary Table 3 – Results from Causal Mediating Analysis (social support, education, exercise)* | | | | | | | | | | | | | |
| --- | --- | --- | --- | --- | --- | --- | --- | --- | --- | --- | --- | --- | --- |
|  |  | **Total effect** | | | **ACME** | | | **ADE** | | | **Prop. mediated** | | |
| Exposure | **Mediator** | **Coef** | **LCI** | **UCI** | **Coef** | **LCI** | **UCI** | **Coef** | **LCI** | **UCI** | **Coef** | **LCI** | **UCI** |
| No Accommodation help | Fatigue VAS | 0.15 | 0.05 | 0.26 | 0.02 | 0.00 | 0.04 | 0.13 | 0.03 | 0.23 | 0.13 | -0.02 | 0.39 |
|  | Pain VAS | 0.15 | 0.05 | 0.26 | 0.00 | -0.02 | 0.02 | 0.15 | 0.04 | 0.25 | 0.02 | -0.14 | 0.18 |
|  | Depression (AIMS) | 0.15 | 0.05 | 0.26 | 0.02 | 0.00 | 0.04 | 0.13 | 0.03 | 0.23 | 0.12 | -0.04 | 0.40 |
|  | Anxiety (AIMS) | 0.15 | 0.05 | 0.25 | 0.01 | -0.01 | 0.03 | 0.14 | 0.04 | 0.24 | 0.04 | -0.11 | 0.21 |
| No Financial help | Fatigue VAS | 0.13 | 0.05 | 0.22 | 0.02 | 0.00 | 0.04 | 0.12 | 0.03 | 0.21 | 0.13 | -0.02 | 0.39 |
|  | Pain VAS | 0.13 | 0.05 | 0.23 | 0.00 | -0.02 | 0.02 | 0.13 | 0.05 | 0.22 | 0.00 | -0.18 | 0.14 |
|  | Depression (AIMS) | 0.13 | 0.04 | 0.22 | 0.02 | 0.00 | 0.05 | 0.11 | 0.02 | 0.20 | 0.16 | 0.02 | 0.53 |
|  | Anxiety (AIMS) | 0.13 | 0.04 | 0.22 | 0.01 | 0.00 | 0.03 | 0.12 | 0.03 | 0.21 | 0.10 | 0.00 | 0.35 |
| No family contact | Fatigue VAS | 0.14 | 0.03 | 0.25 | 0.03 | 0.00 | 0.06 | 0.11 | 0.00 | 0.22 | 0.20 | 0.01 | 0.85 |
|  | Pain VAS | 0.14 | 0.02 | 0.25 | 0.01 | -0.01 | 0.04 | 0.12 | 0.01 | 0.24 | 0.09 | -0.08 | 0.45 |
|  | Depression (AIMS) | 0.13 | 0.02 | 0.25 | 0.04 | 0.02 | 0.07 | 0.09 | -0.02 | 0.21 | 0.32 | 0.09 | 1.61 |
|  | Anxiety (AIMS) | 0.14 | 0.02 | 0.25 | 0.02 | 0.00 | 0.05 | 0.11 | 0.00 | 0.23 | 0.17 | 0.02 | 0.76 |
| Not married / co-habiting | Fatigue VAS | -0.05 | -0.13 | 0.04 | 0.00 | -0.02 | 0.02 | -0.05 | -0.13 | 0.04 | 0.01 | -1.75 | 2.10 |
|  | Pain VAS | -0.05 | -0.13 | 0.04 | 0.01 | -0.01 | 0.03 | -0.05 | -0.14 | 0.04 | -0.07 | -2.30 | 1.73 |
|  | Depression (AIMS) | -0.05 | -0.13 | 0.04 | 0.01 | -0.01 | 0.04 | -0.06 | -0.14 | 0.03 | -0.17 | -3.91 | 2.89 |
|  | Anxiety (AIMS) | -0.05 | -0.13 | 0.04 | 0.00 | -0.02 | 0.02 | -0.05 | -0.13 | 0.04 | -0.01 | -1.70 | 1.26 |
| Low Education | Fatigue VAS | 0.05 | 0.02 | 0.07 | 0.01 | 0.01 | 0.02 | 0.04 | 0.01 | 0.06 | 0.23 | 0.11 | 0.58 |
|  | Pain VAS | 0.05 | 0.02 | 0.07 | 0.01 | 0.00 | 0.02 | 0.04 | 0.01 | 0.06 | 0.21 | 0.08 | 0.61 |
|  | Depression (AIMS) | 0.05 | 0.02 | 0.07 | 0.01 | 0.01 | 0.02 | 0.04 | 0.01 | 0.06 | 0.25 | 0.10 | 0.66 |
|  | Anxiety (AIMS) | 0.05 | 0.02 | 0.07 | 0.01 | 0.00 | 0.01 | 0.04 | 0.02 | 0.07 | 0.12 | 0.03 | 0.37 |
| No exercise | Fatigue VAS | 0.18 | 0.10 | 0.25 | 0.03 | 0.01 | 0.05 | 0.14 | 0.07 | 0.23 | 0.18 | 0.08 | 0.37 |
|  | Pain VAS | 0.18 | 0.10 | 0.26 | 0.01 | 0.00 | 0.03 | 0.17 | 0.09 | 0.24 | 0.06 | -0.01 | 0.17 |
|  | Depression (AIMS) | 0.17 | 0.10 | 0.25 | 0.03 | 0.01 | 0.05 | 0.15 | 0.07 | 0.22 | 0.16 | 0.06 | 0.33 |
|  | Anxiety (AIMS) | 0.18 | 0.10 | 0.25 | 0.02 | 0.01 | 0.04 | 0.15 | 0.08 | 0.23 | 0.13 | 0.04 | 0.28 |
| *ACME = average causal mediated effect, ADE = average direct effect, AIMS = Arthritis Impact Measurement Scale, LCI = lower confidence interval, prop. = proportion, UCI = upper confidence interval, VAS = visual analogue scale* | | | | | | | | | | | | | |

| *Supplementary Table 4 – Results from Causal Mediating Analysis (Financial situation)* | | | | | | | | | | | | | |
| --- | --- | --- | --- | --- | --- | --- | --- | --- | --- | --- | --- | --- | --- |
|  |  | **Total effect** | | | **ACME** | | | **ADE** | | | **Prop. mediated** | | |
| Exposure | **Mediator** | **Coef** | **LCI** | **UCI** | **Coef** | **LCI** | **UCI** | **Coef** | **LCI** | **UCI** | **Coef** | **LCI** | **UCI** |
| Low personal income | Fatigue VAS | 0.12 | 0.02 | 0.21 | 0.03 | 0.01 | 0.06 | 0.09 | -0.01 | 0.18 | 0.25 | 0.06 | 1.14 |
|  | Pain VAS | 0.11 | 0.02 | 0.20 | 0.03 | 0.01 | 0.05 | 0.08 | -0.01 | 0.18 | 0.25 | 0.08 | 1.21 |
|  | Depression (AIMS) | 0.12 | 0.03 | 0.21 | 0.04 | 0.02 | 0.06 | 0.08 | -0.02 | 0.17 | 0.33 | 0.11 | 1.34 |
|  | Anxiety (AIMS) | 0.12 | 0.02 | 0.21 | 0.02 | 0.00 | 0.04 | 0.10 | 0.00 | 0.19 | 0.17 | 0.03 | 0.79 |
| Not home owner | Fatigue VAS | 0.06 | -0.02 | 0.14 | 0.01 | -0.01 | 0.03 | 0.05 | -0.03 | 0.13 | 0.18 | -1.76 | 1.61 |
|  | Pain VAS | 0.06 | -0.02 | 0.14 | 0.01 | -0.01 | 0.02 | 0.05 | -0.03 | 0.14 | 0.09 | -0.98 | 0.99 |
|  | Depression (AIMS) | 0.06 | -0.02 | 0.14 | 0.02 | 0.00 | 0.04 | 0.04 | -0.04 | 0.12 | 0.32 | -2.75 | 3.25 |
|  | Anxiety (AIMS) | 0.06 | -0.02 | 0.14 | 0.02 | 0.00 | 0.03 | 0.04 | -0.04 | 0.13 | 0.21 | -2.24 | 1.87 |
| No show / cinema trips | Fatigue VAS | 0.14 | 0.06 | 0.22 | 0.03 | 0.01 | 0.05 | 0.11 | 0.03 | 0.20 | 0.18 | 0.06 | 0.48 |
|  | Pain VAS | 0.14 | 0.06 | 0.23 | 0.02 | 0.01 | 0.04 | 0.12 | 0.04 | 0.20 | 0.16 | 0.05 | 0.43 |
|  | Depression (AIMS) | 0.14 | 0.06 | 0.22 | 0.04 | 0.02 | 0.07 | 0.09 | 0.01 | 0.18 | 0.33 | 0.14 | 0.82 |
|  | Anxiety (AIMS) | 0.14 | 0.06 | 0.22 | 0.02 | 0.01 | 0.04 | 0.12 | 0.04 | 0.21 | 0.13 | 0.03 | 0.37 |
| No holiday | Fatigue VAS | 0.15 | 0.08 | 0.23 | 0.02 | 0.00 | 0.04 | 0.13 | 0.05 | 0.21 | 0.14 | 0.03 | 0.33 |
|  | Pain VAS | 0.15 | 0.08 | 0.23 | 0.01 | -0.01 | 0.02 | 0.15 | 0.07 | 0.23 | 0.04 | -0.05 | 0.15 |
|  | Depression (AIMS) | 0.15 | 0.07 | 0.23 | 0.03 | 0.01 | 0.05 | 0.12 | 0.04 | 0.20 | 0.21 | 0.08 | 0.46 |
|  | Anxiety (AIMS) | 0.15 | 0.07 | 0.23 | 0.02 | 0.01 | 0.04 | 0.13 | 0.05 | 0.21 | 0.13 | 0.04 | 0.32 |
| Low job level | Fatigue VAS | 0.11 | 0.05 | 0.16 | 0.01 | 0.00 | 0.02 | 0.10 | 0.04 | 0.16 | 0.08 | -0.04 | 0.23 |
|  | Pain VAS | 0.11 | 0.05 | 0.16 | 0.02 | 0.01 | 0.03 | 0.09 | 0.03 | 0.15 | 0.16 | 0.06 | 0.41 |
|  | Depression (AIMS) | 0.11 | 0.06 | 0.17 | 0.02 | 0.00 | 0.03 | 0.10 | 0.04 | 0.15 | 0.13 | 0.02 | 0.35 |
|  | Anxiety (AIMS) | 0.11 | 0.06 | 0.17 | 0.00 | -0.01 | 0.02 | 0.11 | 0.05 | 0.16 | 0.02 | -0.09 | 0.14 |
| Not working | Fatigue VAS | 0.03 | -0.06 | 0.11 | 0.00 | -0.02 | 0.02 | 0.03 | -0.05 | 0.12 | -0.01 | -3.34 | 2.25 |
|  | Pain VAS | 0.03 | -0.06 | 0.11 | 0.01 | -0.01 | 0.02 | 0.02 | -0.07 | 0.11 | 0.10 | -2.55 | 3.46 |
|  | Depression (AIMS) | 0.03 | -0.06 | 0.12 | 0.00 | -0.02 | 0.02 | 0.02 | -0.06 | 0.11 | 0.04 | -2.15 | 2.29 |
|  | Anxiety (AIMS) | 0.03 | -0.06 | 0.12 | 0.00 | -0.02 | 0.02 | 0.03 | -0.06 | 0.12 | 0.01 | -2.29 | 1.91 |
| Low family income | Fatigue VAS | 0.11 | 0.03 | 0.19 | 0.03 | 0.01 | 0.05 | 0.08 | 0.01 | 0.16 | 0.24 | 0.08 | 0.75 |
|  | Pain VAS | 0.11 | 0.03 | 0.19 | 0.03 | 0.01 | 0.05 | 0.08 | 0.00 | 0.16 | 0.25 | 0.08 | 0.86 |
|  | Depression (AIMS) | 0.11 | 0.03 | 0.19 | 0.04 | 0.02 | 0.06 | 0.07 | -0.01 | 0.15 | 0.37 | 0.14 | 1.21 |
|  | Anxiety (AIMS) | 0.11 | 0.03 | 0.19 | 0.02 | 0.00 | 0.04 | 0.09 | 0.01 | 0.17 | 0.17 | 0.04 | 0.58 |
| *ACME = average causal mediated effect, ADE = average direct effect, AIMS = Arthritis Impact Measurement Scale, LCI = lower confidence interval, prop. = proportion, UCI = upper confidence interval, VAS = visual analogue scale* | | | | | | | | | | | | | |

| *Supplementary Table 5 – Baseline characteristics of the validation datasets: NOAR and ERAN* | | | | | | | | |
| --- | --- | --- | --- | --- | --- | --- | --- | --- |
|  | **NOAR** | | | | **ERAN** | | | |
| Variable | **Total NOAR cohort,**  **Mean (SD) / N (%)** | **Excess disability,**  **Mean (SD) / N (%)** | **No excess disability,**  **Mean (SD) / N (%)** | **p** | **Total ERAN cohort,**  **Mean (SD) / N (%)** | **Excess disability,**  **Mean (SD) / N (%)** | **No excess disability,**  **Mean (SD) / N (%)** | **p** |
| N | 416 | 166 (39.9%) | 250 (60.1%) |  | 386 | 198 (51.3%) | 188 (48.7%) |  |
| *Demographics* |  |  |  |  |  |  |  |  |
| Age, years | 57.3 (14.2) | 59.9 (14.3) | 55.5 (13.9) | 0.002† | 57.1 (13.1) | 58.2 (13.3) | 56.0 (12.8) | 0.108† |
| Women, N(%) | 276 (66.3%) | 108 (65.1%) | 168 (67.2%) | 0.729‡ | 275 (71.2%) | 154 (77.8%) | 121 (64.4%) | 0.005‡ |
| Symptom duration, months | 8.0 (5.2) | 8.3 (5.4) | 7.7 (5.1) | 0.302† | 10.2 (5.1) | 9.9 (5.3) | 10.5 (4.8) | 0.247† |
| *PROMs* |  |  |  |  |  |  |  |  |
| NOAR: Pain VAS / ERAN: SF36 pain | 46.6 (27.2) | 57.1 (26.8) | 39.6 (25.2) | <0.001† | 40.2 (23.3) | 39.0 (24.2) | 41.4 (22.2) | 0.323† |
| NOAR: Fatigue VAS / ERAN: SF36 vitality | 49.9 (28.3) | 59.0 (26.4) | 43.9 (27.9) | <0.001† | 39.4 (20.7) | 39.0 (21.1) | 39.8 (20.4) | 0.695† |
| NOAR: AIMS anxiety / ERAN: SF36MH] | 4.4 (2.1) | 4.8 (2.1) | 4.1 (2.0) | <0.001† | 64.0 (19.5) | 63.4 (20.4) | 64.7 (18.5) | 0.500† |
| AIMS depression | 3.3 (2.0) | 4.0 (2.1) | 2.9 (1.9) | <0.001† | - | - | - | - |
| HAQ | 1.2 (0.7) | 1.7 (0.6) | 0.9 (0.6) | <0.001† | 1.3 (0.7) | 1.4 (0.8) | 1.2 (0.5) | 0.007† |
| *Disease activity* |  |  |  |  |  |  |  |  |
| DAS28 [NOAR=CRP, ERAN=ESR] | 4.1 (1.3) | 4.4 (1.4) | 4.0 (1.2) | 0.002† | 4.7 (1.3) | 4.8 (1.2) | 4.6 (1.3) | 0.128† |
| DAS28-2c [NOAR=CRP, ERAN=ESR] | 3.3 (1.5) | 3.4 (1.7) | 3.3 (1.5) | 0.390† | 3.2 (1.2) | 3.3 (1.2) | 3.1 (1.3) | 0.111† |
| Swollen joint count (28) | 5.1 (5.4) | 5.7 (6.2) | 4.7 (4.8) | 0.064† | 6.1 (5.3) | 6.4 (5.1) | 5.9 (5.5) | 0.317† |
| Tender joint count (28) | 7.6 (7.7) | 9.6 (8.8) | 6.3 (6.5) | <0.001† | 8.1 (6.8) | 8.4 (6.6) | 7.8 (7.1) | 0.418† |
| CRP [NOAR] / ESR [ERAN] | 20.7 (58.3) | 18.7 (23.1) | 22.0 (72.8) | 0.506† | 31.5 (24.0) | 32.3 (24.0) | 30.7 (24.1) | 0.517† |
| *† t-test. ‡ chi^2^ test*  *AIMS = Arthritis Impact Measurement Scales, CRP = C-reactive protein, DAS28 = Disease Activity Score 28, DAS28-2c = two-component Disease Activity Score, ERAN = Early Rheumatoid Arthritis Network, ESR = erythrocyte sedimentation rate, HAQ = Health Assessment Questionnaire, N = number, NOAR = Norfolk Arthritis Register, SD = standard deviation, VAS = visual analogue scale* | | | | | | | | |

| *Supplementary table 6 – NOAR and ERAN work-related and deprivation variables, total cohort and stratified by excess disability groups* | | | | |
| --- | --- | --- | --- | --- |
| Work / deprivation variable | **Total cohort, N(%)** | **Excess**  **disability, N(%)** | **No excess disability, N(%)** | **OR of excess disability membership (95% CI)** |
| NOAR |  |  |  |  |
| Job status: |  |  |  |  |
| Working, N(%) | 164 (39.4%) | 45 (27.1%) | 119 (47.6%) | Ref. |
| Not working (at home, unemployed, student, disabled, long-term illness), N(%) | 73 (17.5%) | 36 (21.7%) | 37 (14.8%) | 2.59 (1.45, 4.62) |
| Retired, N(%) | 179 (43.0%) | 85 (51.2%) | 94 (37.6%) | 1.64 (0.87, 3.12) |
| Job level |  |  |  |  |
| Low, N(%) | 102 (24.5%) | 47 (28.3%) | 55 (22.0%) | 0.98 (0.60, 1.62) |
| Medium, N(%) | 172 (41.3%) | 79 (47.6%) | 93 (37.2%) | Ref. |
| High, N(%) | 142 (34.1%) | 40 (24.1%) | 102 (40.8%) | 0.45 (0.28, 0.72) |
| ERAN |  |  |  |  |
| Job status: |  |  |  |  |
| Working, N(%) | 183 (47.4%) | 89 (44.9%) | 94 (50.0%) | Ref. |
| Not working (at home, unemployed, student, disabled, long-term illness), N(%) | 66 (17.1%) | 35 (17.7%) | 31 (16.5%) | 1.08 (0.61, 1.93) |
| Retired, N(%) | 137 (35.5%) | 74 (37.4%) | 63 (33.5%) | 0.78 (0.41, 1.49) |
| IMD 2007 decile, mean (SD) | 5.6 (2.5) | 5.8 (2.5) | 5.4 (2.6) | 1.06 (0.97, 1.15) |
| *OR adjusted for age and gender*  *CI = confidence interval, ERAN = Early Rheumatoid Arthritis Network, IMD = Index of Multiple Deprivation, OR = odds ratio, N = number, NOAR = Norfolk Arthritis Register, SD = standard deviation* | | | | |

| *Supplementary table 7 – Results of SEM / path analyses within the NOAR and ERAN datasets* | | | | |
| --- | --- | --- | --- | --- |
|  | **NOAR** | | **ERAN** | |
|  | **Employment status** | **Job status** | **Employment status** | **IMD 2007** |
| *Mediation SEM* | **Standardised β**  **(95% CI)** | **Standardised β**  **(95% CI)** | **Standardised β (95% CI)** | **Standardised β (95% CI)** |
| Total Effect on high HAQ membership | 0.176 (0.067, 0.285) | 0.158 (0.065, 0.250) | -0.015 (-0.130, 0.101) | 0.069 (-0.031, 0.168) |
| Direct effect | 0.086 (-0.021, 0.193) | 0.108 (0.020, 0.195) | -0.023 (-0.140, 0.093) | 0.079 (-0.021, 0.180) |
| Proportion of total effect unexplained by PROMs | 49% (-24, 76) | 68% (29, 91) | - | - |
| Indirect effect through pain | 0.042 (0.009, 0.074) | 0.026 (0.000, 0.052) | 0.011 (-0.006, 0.028) | -0.011 (-0.027, 0.005) |
| Proportion mediated through pain | 24% (7, 69) | 16% (2, 42) | - | - |
| Indirect effect through fatigue | 0.021 (-0.001, 0.042) | 0.010 (-0.005, 0.024) | -0.002 (-0.018, 0.014) | 0.001 (-0.007, 0.009) |
| Proportion mediated through fatigue | 12% (1, 32) | 6% (-2, 19) | - | - |
| Indirect effect through depression | 0.027 (-0.003, 0.058) | 0.014 (-0.004, 0.032) | -0.000 (-0.006, 0.006) | -0.001 (-0.016, 0.014) |
| Proportion mediated through depression | 15% (-1, 50) | 9% (-1, 28) | - | - |
| Indirect effect through anxiety | 0.001 (-0.004, 0.006) | 0.000 (-0.011, 0.012) | - | - |
| Proportion mediated through anxiety | 1% (-5, 8) | 0% (-9, 10) | - | - |
| *CI = confidence interval, ERAN = Early Rheumatoid Arthritis Network, HAQ = Health Assessment Questionnaire, NOAR = Norfolk Arthritis Register, PROMs = patient reported outcome measures, SEM = structural equation model* | | | | |

**References**

1. Gwinnutt J, Norton S, Hyrich K, et al. Exploring the disparity between inflammation and disability in the ten year outcomes of people with rheumatoid arthritis. *Rheumatology (Oxford)* 2022;Online ahead of print
